# Efficacy of positron emission tomography in diagnosis of lateral lymph node metastases in patients with rectal Cancer: a retrospective study

**DOI:** 10.1186/s12885-021-08278-6

**Published:** 2021-05-08

**Authors:** Ryohei Yukimoto, Mamoru Uemura, Takahiro Tsuboyama, Tsuyoshi Hata, Shiki Fujino, Takayuki Ogino, Norikatsu Miyoshi, Hidekazu Takahashi, Taishi Hata, Hirofumi Yamamoto, Tsunekazu Mizushima, Akira Kida, Mamoru Furuyashiki, Yuichiro Doki, Hidetoshi Eguchi

**Affiliations:** 1 Department of Gastroenterological Surgery, Graduate School of Medicine, Osaka University, 2-2 Yamada-oka, Suita City, Osaka, 565-0871 Japan; 2 Department of Radiology, Graduate School of Medicine, Osaka University, Osaka, Japan; 3 Department of Radiology, Jinsenkai MI Clinic, Toyonaka, Osaka, Japan

**Keywords:** Positron emission tomography, Lateral pelvic lymph node, Rectal cancer, Maximum standardized uptake value; metastases

## Abstract

**Background:**

The presence of lateral pelvic lymph node (LLN) metastasis is an essential prognostic factor in rectal cancer patients. Thus, preoperative diagnosis of LLN metastasis is clinically important to determine the therapeutic strategy. The aim of this study was to evaluate the efficacy of preoperative positron emission tomography/computed tomography (PET/CT) in the diagnosis of LLN metastasis.

**Methods:**

Eighty-four patients with rectal cancer who underwent LLN dissection at Osaka University were included in this study. The maximum standardized uptake value (SUV_max_) of the primary tumor and LLN were preoperatively calculated using PET/CT. Simultaneously, the short axis of the lymph node was measured using multi-detector row computed tomography (MDCT). The presence of metastases was evaluated by postoperative pathological examination.

**Results:**

Of the 84 patients, LLN metastases developed in the left, right, and both LLN regions in 6, 7, and 2 patients, respectively. The diagnosis of the metastases was predicted with a sensitivity of 82%, specificity of 93%, positive predictive value of 58%, negative predictive value of 98%, false positive value of 7%, and false negative value of 18% when the cutoff value of the LLN SUV_max_ was set at 1.5. The cutoff value of the short axis set at 7 mm on MDCT was most useful in diagnosing LLN metastases, but SUV_max_ was even more useful in terms of specificity.

**Conclusions:**

The cutoff value of 1.5 for lymph node SUV_max_ in PET is a reasonable measure to predict the risk of preoperative LLN metastases in rectal cancer patients.

## Background

The presence of lateral pelvic lymph node (LLN) metastasis is an important prognostic factor in patients with lower rectal cancer [1]. The treatment strategy for LLN differs between Japan, Europe, and North America. In Europe and North America, LLN metastases are considered a metastatic disease, and total mesorectal excision (TME) with preoperative radiotherapy and chemotherapy is commonly performed [2, 3]. Conversely, in Japan, LLN metastases are considered a local metastasis, and TME with lateral lymph node dissection (LLND) is performed as the standard surgical procedure for advanced lower rectal cancer.

Several previous reports have indicated the efficacy of LLND and its prognostic impact [4–7]. However, according to some reports, this surgical procedure has several potential disadvantages such as hemorrhage, prolonged surgical time, and risk of complications such as dysuria and sexual dysfunction [8, 9]. Considering these potential complications, accurate preoperative prediction of LLN metastasis is required to identify the patients who are suitable for LLND. Currently, the preoperative diagnosis of lymph node metastases is mainly based on their size on computed tomography (CT) or magnetic resonance imaging (MRI) scans. A study conducted by the Japan Clinical Oncology Group (JCOG 0212) set the cutoff value for the short axis of a lymph node at 10 mm for the prediction of LLN metastases [9]. To date, several studies have focused on preoperative diagnosis of LLN metastases, and several diagnostic criteria have been indicated. These studies employed cutoff values between 5 mm and 10 mm, but the results varied [10–12]. Until now, no definitive conclusions have been drawn regarding the optimal cutoff value.

^18^F-fluorodeoxyglucose positron emission tomography/computed tomography (^18^F-FDG PET/CT), a technique that reveals the biological variability of tumors, has been widely used to preoperatively evaluate rectal cancer in recent years [13, 14]. This study aimed to assess the effectiveness of PET/CT in the preoperative diagnosis of LLN metastases. Furthermore, we investigated the optimal cutoff value for the prediction of such metastases.

## Methods

### Patients

This retrospective study aimed to assess the effectiveness of ^18^F-FDG PET/CT in the preoperative diagnosis of LLN metastases. We included patients with rectal cancer who underwent elective surgery with LLND at Osaka University (Suita, Japan) from January 2011 to December 2019. The patients were excluded from the study if 1) they had not undergone preoperative ^18^F-FDG PET/CT scans (*n* = 13), 2) they had been diagnosed with locally recurrent rectal cancer (*n* = 11), 3) they had been diagnosed as squamous cell carcinoma (*n* = 2) and 4) the patient who underwent unilateral selective LLND (*n* = 2).

#### The criteria of LLND

LLND is indicated for patients with lower rectal cancer at or below the peritoneal reflection when they have cT3 or T4 rectal cancer or when positive lymph nodes are suspected. Basically, bilateral lateral lymph node dissection (internal iliac and obturator nodes dissection) was performed in the patients who met the criteria of LLND.

#### The criteria of neoadjuvant treatment in rectal cancer

Neoadjuvant treatment is indicated for patients with lower rectal cancer at or below the peritoneal reflection when they have cT3 or T4 rectal cancer or when positive lymph nodes are suspected, those determined to be able to tolerate neoadjuvant therapy based on their age and performance status (PS). In patients who underwent neoadjuvant chemotherapy or neoadjuvant chemoradiotherapy, imaging tests were performed to evaluate the treatment response; however, the indication for LLND was determined based on the diagnosis prior to the start of treatment.

### Positron emission tomography/computed tomography

Briefly, ^18^F-FDG PET/CT scans were obtained using the Discovery 710 (GE Health, Japan). The PET parameter included was the maximum standardized uptake value (SUV_max_).

Three-dimensional data acquisition was initiated 60 min after the injection of 4.8 MBq/kg of ^18^F-FDG. The SUV_max_ in the region of interest (ROI) was used as a representative value for the assessment of FDG uptake in the lesion.

### Multi-detector row computed tomography

Multi-detector row computed tomography (MDCT) scanning was performed at the same time as PET/CT. The MDCT parameters were as follows: tube voltage 120 kV, tube current 10–320 mA, automatic exposure control in the x, y, and z planes with a noise index of 11.0, rotation speed 0.6 s/r, helical pitch 17.5 mm/r, and slice thickness 0.625 mm. The reconstruction intervals were set to 0.5 mm. For the contrast-enhanced MDCT images, a nonionic contrast agent with an iodine concentration of 350 mg/mL (Optiray, Guerbet Japan, Osaka, Japan) was infused at a flow rate of 4.0 mL/s followed by saline at the same rate during arterial phase scanning with a dual-head injector (Stellant, Medrad, Indianola, PA, USA). The volume of injected contrast agent was 100 mL for patients weighing > 49 kg, and 2.0 mL/kg for patients weighing < 50 kg. To determine the arterial phase scan delay, a test injection with 10 mL of contrast agent and 10 mL of saline was performed at the same rate [15]. The imaging examinations using PET, followed by MDCT were performed on the same day.

### Evaluation of diagnostic performance

The diagnostic performance of SUV_max_ of PET was analyzed in the right and left LLN regions. The largest short axes of the lymph nodes in the left and right LLN regions were measured, and their SUV_max_ were also measured.

**Fig. 1:**
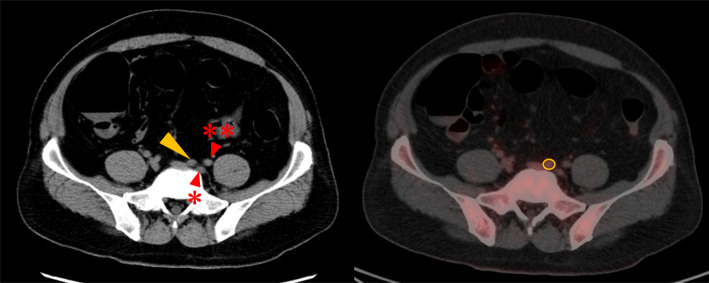
Multidetector computed tomography detecting the largest short axis of the lymph node in the left lateral lymph node region (left image), and measurement of SUVmax using region of interest on PET-CT (right image). Internal iliac artery (red arrow *), external iliac artery (red arrow **), the lateral lymph node of internal iliac region #263 (yellow arrow), the region of interest (yellow circle)

Specifically, the lymph node with the largest diameter on the short axis in the target area was identified using multidetector computed tomography (MDCT), and the maximum standardized uptake value (SUVmax) of the detected lymph node was measured by overlaying images obtained from PET-CT imaging (Fig. 1).

The short axes of lymph nodes in both regions were measured using the Universal Viewer Ver 6.0 (GE Healthcare) and ROI analysis was performed. The SUV_max_ of the largest short axis of the LLN and the primary tumor were measured (in the axial plane). These measured values were compared and evaluated with the postoperative pathology.

The receiver operating characteristic (ROC) curve was used to determine the optimal cutoff values of the lymph node SUV_max_ for the prediction of metastasis. The cutoff values were calculated with the highest sensitivity and the highest specificity located on the ROC curve at the highest point on the vertical axis and furthest point to the left on the horizontal axis.

### Statistical analysis

All analyses were performed using JMP Pro 14 for Windows (SAS Institute, USA). The pathological status and SUV_max_ of the LLN were collected to generate the ROC curve. Furthermore, the corresponding areas under the ROC curve (AUC) were calculated. The optimal cutoff was detected using Youden index. The sensitivity, specificity, positive predictive value (PPV), negative predictive value (NPV), false positive (FP), and false negative (FN) were determined based on the comparison of the PET/CT images using a cutoff value of 1.5 for lymph node SUV_max_ and short axis (10 and 7 mm) with histological diagnosis. To compare the sensitivity and specificity for the evaluation of two diagnostic tests, McNemar test was carried out. Results were considered statistically significant when *p* < 0.05 was obtained.

## Results

In total, 112 patients underwent LLND at Osaka University during January 2011–December 2019. Of these, we excluded 13 patients who did not undergo preoperative ^18^F-FDG PET/CT scans, 11 who were diagnosed with locally recurrent rectal cancer, 2 who were diagnosed as squamous cell carcinoma and 2 who underwent unilateral selective LLND. Finally, 84 patients (52 men and 32 women) who underwent LLND for primary rectal cancer were included in this study (Table 1).

**Table 1:** Clinical and pathological characteristics of the patients and preoperative PET results

Characteristics	*N* = 84
Age (years)	
Median/Range	62 (27–83)
Sex (male/female)	53 (63%)/31 (37%)
Preoperative chemotherapy (+/−)	66 (79%)/18 (21%)
Preoperative radiation therapy (+/−)	3 (4%)/81 (96%)
Tumor differentiation (well differentiated tubular adenocarcinoma/moderately differentiated tubular adenocarcinoma/mucinous adenocarcinoma)	43/37/4
Pathological T stage (T0/Tis/T1/T2/T3/T4)	6/1/8/21/37/11
Pathological N stage (N0/N1/N2/)	51/18/15
LLN metastasis (+/−)	15 (18%)/69 (82%)
The median number of resected LLND Right side / Left side	9 (6–12)/ 10 (7–13)
Location of LLN metastasis (263R/263 L/273R/273 L/283R/283 L/293R/293 L)	4/5/0/0/6/5/0/1
Median and variance of short axis diameter for lateral lymph nodes Positive [median, variance]/ negative [median, variance]	[10, 10.31/ 4.8, 3.8]
Median and variance of SUVmax for lateral lymph nodes positive [median, variance]/ negative [median, variance]	[1.9, 17.7 / 0.91, 0.24]
Pathological stage 0/I/II/III/IV	6/22/20/22/14
Primary tumor SUV_max_	14.2 ± 6.89
LLN SUV_max_	1.4 ± 1.70

*LLN* lateral pelvic lymph node, *PET* positron emission tomography, *SUV*_*max*_ maximum standardized uptake value; 263, internal iliac region; 273, common iliac region; 283, obturator region; 293, external iliac region

LLND was performed in 84 patients who underwent TME: 37 with low anterior resection, 24 with intersphincteric resection, 19 with abdominoperineal resection, and 4 with total pelvic exenteration.

### Incidence of lateral lymph node metastasis

LLN metastasis was identified in 15 patients (18%) based on histopathological examination of whom, 13 had unilateral lymph node metastasis and 2 had bilateral lymph node metastasis. LPLN metastases had developed in the right region in 6 patients (2 in the right internal iliac region, 3 in the right obturator region, 1 in the right internal iliac region and right obturator region), left region in 7 patients (2 in the left internal iliac region,3 in the left obturator region, 1 in the left external iliac region,1 in the left internal iliac region and left obturator region), and bilaterally in 2 patients (1 in the right internal iliac region, right obturator region, left internal iliac region, and left obturator region and 1 in the right obturator region and left internal iliac region).

The characteristics of the 17 lateral lymph nodes that were positive for metastasis are shown in Table 2.

**Table 2:** Characteristics of the 17 lymph nodes positive for metastasis

	Location	Size	SUVmax	Tumor initial staging	Neoadjuvant therapy
No.1	263R	14.8	4.19	II	–
No.2^a^	263R	10.6	9.56	III	+
No.3^a^	283 L	15.2	10.28	III	+
No.4^b^	263R	15	11.13	III	+
No.5^b^	263 L	10.2	13.66	III	+
No.6	283R	9	6.65	III	+
No.7	283R	10.1	1.85	IV	–
No.8	283R	5.1	0.78	III	+
No.9	263R	9.8	1.85	III	+
No.10	283R	10.3	2.20	III	+
No.11	263 L	6.5	1.82	III	–
No.12	263 L	6.6	1.58	III	+
No.13	283 L	6.5	1.26	II	+
N0.14	283 L	11.2	2.01	II	+
N0.15	283 L	9	1.54	III	+
No.16	293 L	4.8	1.13	III	–
N0.17	263 L	10	1.91	III	–

263, internal iliac region; 283, obturator region; 293, external iliac region

^a^: Same Case 1, ^b^: Same case 2

We found that the short axes of the LLN and SUVmax were independent predictors for LLN metastasis on a logistic regression analysis (*P* < 0.05).

### Cutoff value evaluation using ROC curve analysis

**Fig. 2:**
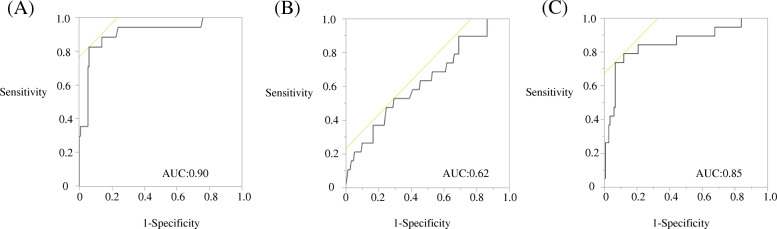
**a**. Receiver operating characteristic (ROC) curve of the maximum standardized uptake value (SUV_max_) in the lateral pelvic lymph nodes as a predictor of pathological metastasis for lateral pelvic lymph nodes. **b**. ROC curve of the SUV_max_ in the primary tumor as a predictor of pathological metastasis for lateral pelvic lymph nodes. **c**. ROC curve of the SUV_max_ in the lateral pelvic lymph nodes/primary tumor as a predictor of pathological metastasis for lateral pelvic lymph nodes

The ROC curve analysis for per-patient prediction of the lymph node status is shown in Fig. 2. ROC curve analysis of the LLN SUV_max_, primary SUV_max_, and LLN SUV_max_/primary SUV_max_ ratio confirmed that LLN SUV_max_ was the best predictor for metastasis while primary SUV_max_ was the poorest among the three. The AUC of the LLN SUV_max_ was 0.90.

The best cutoff value of the LLN SUV_max_ was found to be 1.5, based on the ROC curve analysis.

**Fig. 3:**
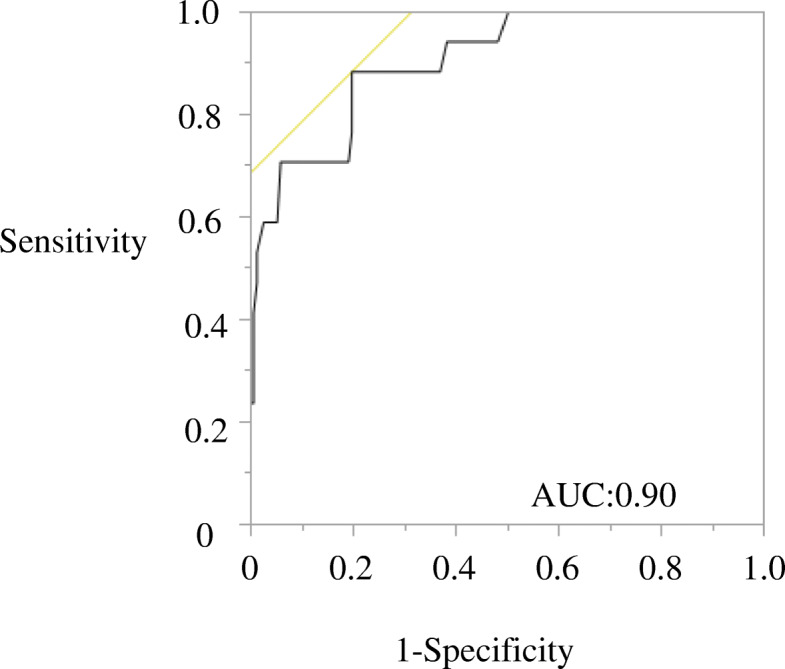
Receiver operating characteristic curves of the short axes of lateral pelvic lymph nodes as a predictor of pathological metastasis for lateral pelvic lymph nodes

Among the 84 patients (168 lymph nodes examined in total), the pathology examination results were positive in 15 patients (17 lymph nodes). Of these, the PET/CT results were negative in three patients (3 lymph nodes) when the cutoff value of the LLN was set at 1.5 **(**Table 3). Furthermore, the MDCT results were negative in 5 patients (5 lymph nodes) when the cutoff value of the short axis of the lymph node was set at 7 mm and in 6 patients (6 lymph nodes) when the cutoff value of the short axis of the lymph node was set at 10 mm (Tables 4, 5). The ROC curve analysis for per-patient prediction of the short axis of the lymph node is shown in Fig. 3. The best cutoff value of the short axis of the lymph node was found to be 7 mm based on the ROC curve analysis.

**Table 3:** Prediction of metastases using PET/CT based on histopathological diagnosis

	Histopathological diagnosis		
	Negative	Positive	Total
PET			
Negative	141	3	144
Positive	10	14	24
Total	151	17	168

The cutoff value of the lateral lymph node SUV_max_ was set at 1.5

*PET* positron emission tomography

**Table 4:** Prediction of metastases using MDCT based on histopathological diagnosis

	Histopathological diagnosis		
	Negative	Positive	Total
MDCT			
Negative	124	5	129
Positive	27	12	39
Total	151	17	168

The cutoff value of the short axis of the lymph node was set at 7 mm

*MDCT* Multi-detector row computed tomography

**Table 5:** Prediction of metastases using MDCT based on histopathological diagnosis

	Histopathological diagnosis		
	Negative	Positive	Total
MDCT			
Negative	144	6	150
Positive	7	11	18
Total	151	17	168

The cutoff value of the short axis of the lymph node was set at 10 mm

*MDCT* Multi-detector row computed tomography

### Sensitivity, specificity, PPV, and NPV

The diagnostic performance of PET when the SUV_max_ cutoff value was 1.5 was as follows: sensitivity, 82.4%; specificity, 93.4%; PPV, 58.3%; NPV, 97.9%; FP, 6.6%; FN, 17.6%; and accuracy, 92.3%. Moreover, the diagnostic performance of MDCT with the cutoff value of 10 mm was as follows: sensitivity of 64.7%, specificity of 95.4%, PPV of 61.1%, and NPV 96.0%; FP, 4.6%; FN, 35.3%; and accuracy, 92.3%. At a cutoff value of 7 mm, the corresponding values for sensitivity, specificity, PPV, NPV, FP, FN and accuracy were 70.6, 82.1, 30.8, 96.1, 17.9, 29.4 and 81.0%, respectively (Table 6).

**Table 6:** Sensitivity, specificity, PPV, NPV, FP, and FN using PET/CT and MDCT

	Sensitivity	Specificity	PPV	NPV	FP	FN	Accuracy
SUV_max_ Cutoff value 1.5	82.4%	93.4%	58.3%	97.9%	6.6%	17.6%	92.3%
MDCT Cutoff value 10 mm (short axis)	64.7%	95.4%	61.1%	96.0%	4.6%	35.3%	92.3%
MDCT Cutoff value 7 mm (short axis)	70.6%	82.1%	30.8%	96.1%	17.9%	29.4%	81.0%

The cutoff value of the lateral pelvic lymph node SUV_max_ was set at 1.5 and that of the short axis of the lymph nodes in MDCT was set at 10 mm and 7 mm

*FP* false positive, *FN* false negative, *MDCT* Multi-detector row computed tomography, *NPV* negative predictive value, *PET* positron emission tomography, *PPV* positive predictive value

In patients with LLN metastases, there was no statistically significant difference in sensitivity between the short axis diameters, which set a cutoff value of MDCT and SUV_max_ at 7 mm, and that of PET at 1.5 (*P* = 0.157). In patients without LLN metastases, there was a statistically significant difference in specificity between the short axis diameters, which set a cutoff value of MDCT and SUV_max_ at 7 mm, and that of PET at 1.5 (*P* < 0.01).

## Discussion

In this retrospective study, we evaluated the effectiveness of PET/CT in the preoperative diagnosis of LLN metastasis. Lymph node metastasis is an important prognostic factor in patients with colorectal cancer [1]. In addition to the number of metastases, the sites of metastases also influence the prognosis. Specifically, LLN metastases are strongly associated with the prognosis in patients with rectal cancer [16]. Therefore, in Japan, LLND is regarded as a standard surgical procedure for these patients. However, to determine the necessity of LLND, the prognostic impact of LLN metastasis should be determined. At the same time, the clinical benefit of LLND should be weighed against the possible operation-related morbidities, especially the deterioration of voiding and sexual function due to nerve injury.

The size of the lymph nodes tends to be larger in patients with LLN metastasis than in those without metastasis; thus, the size of the node has been widely used as a reference in diagnosing metastases [5]. Several studies have evaluated the accuracy of the preoperative prediction of metastasis based on the short axis diameter of a lymph node in MDCT or MRI. When the cutoff value of the short axis was set between 5 and 10 mm, the sensitivity, specificity, and accuracy rate were 50–85%, 41–75%, and 51–86%, respectively, showing high variability. Thus, it is unknown whether the patients with LLN metastasis are accurately identified before surgery based on the measurement of the short axis diameter of the lymph node. Recently, PET/CT has been widely used for the staging of rectal cancer and determining the treatment strategy in patients with rectal cancer [17]. In particular, SUV_max_ is commonly employed for ^18^F-FDG quantification. However, few studies have evaluated the efficacy of PET in diagnosing LLN metastases in patients with rectal cancer. The AUC was an indicator of the effectiveness of the examination. The present study showed that the AUC was 0.91. Based on the present study, PET-CT is likely to be useful for predicting the presence of LLN metastases and, since NPV is high and FN is low, this is considered to be an effective evaluation method to decide about the need for performing LLND.

When we compared the sensitivity and specificity between diagnostics based on LLN short axis diameter and diagnostic method using the SUV_max_ value of LLN in patients with LLN metastases, we found a statistically significant difference in specificity between the short axis diameters, setting a cutoff value for MDCT at 7 mm and that of and SUV_max_ for PET/CT at 1.5. To select the patients with LLN metastases for LLND, high specificity might be crucial. The results of this study and those of previous studies using CT and MRI for predicting metastases have been compared in Table 7 [8, 10, 11, 18–20]. Compared to the previous studies, the sensitivity was particularly higher and FN rates were lower in this study. We evaluated false negative rate in Table 4. Using SUVmax of 1.5 as the criteria, false negative was 17.6%, which was the lowest among the considered criteria. This indicates that LLN SUV_max_ is highly useful for establishing the indication criteria for LLND. Recently, evaluation of MRI findings, including concentrations, shape, and the short axis of the lymph node has been reported with the goal of improving diagnostic accuracy. In addition, to improve the diagnostic accuracy, other factors suggestive of metastases, such as “an irregular border” and “mixed signal intensity or the presence of a high-intensity nodule within the lymph node,” should be considered. However, the evaluation of the MRI scans is primarily subjective and influenced by the experience of the assessors, making it difficult to establish an objective standard evaluation method [21].

**Table 7:** Comparison of cutoff value, sensitivity, PPV, NPV, and accuracy across previous studies and present study

Author	Year	Number	Modality	Cutoff value	Sensitivity	Specificity	PPV	NPV	Accuracy
Arii et al. [18]	2006	53	CT	7 mm	22%	78%	8%	95%	75%
Arii et al. [18]	2006	53	MRI	7 mm	56%	97%	91%	81%	83%
Fujita et al. [8]	2009	210	CT	5 mm	62%	90%	64%	89%	84%
Akasu et al. [10].	2009	104	MRI	4 mm (short axis)	87%	87%	52%	97%	87%
Akasu et al. [10]	2009	104	MRI	3 mm (short axis)	93%	81%	45%	99%	83%
Matsuoka et al. [11]	2007	51	MRI	5 mm (short axis)	67%	83%	–	–	78%
Ishibe et al. [19]	2016	84	MRI	10 mm (short axis)	43.8%	98.5%	87.5%	88.2%	88.1%
Amano et al. [20].	2019	46	MRI	6 mm (short axis)	35.3%	97%	54.6%	94%	91.3%
Amano et al. [20]	2019	46	CT	6 mm (short axis)	35.3%	100%	100%	96.7%	94%
This study	2020	84	PET	SUV_max_ 1.5	82.4%	93.4%	58.3%	97.9%	92.3%
This study	2020	84	MDCT	10 mm (short axis)	64.7%	95.4%	61.1%	96.0%	92.3%
This study	2020	84	MDCT	7 mm (short axis)	70.6%	82.1%	30.8%	96.1%	81.0%

*CT* computed tomography, *MDCT* Multi-detector row computed tomography, *MRI* magnetic resonance imaging, *NPV* negative predictive value, *PET* positron emission tomography, *PPV* positive predictive value, *SUV*_*max*_ maximum standardized uptake value

The severity of preoperative lymph node metastasis may be underestimated due to slice intervals in CT and MRI scans. However, this may be compensated by PET analysis because it shows the biological variability of the lymph nodes. Considering this, metastases may be suspected in PET when FDG gets accumulated in the lymph nodes. Several studies on the prediction of lymph node metastases in patients with colorectal or rectal cancers have been conducted, in which the cutoff value of SUVmax was set within the range of 1.15–2.5 [22–25]. Although the present study focused only on the LLN, no significant difference was observed in the cutoff values when comparing the results of our study with those of other studies. However, the limitation of these studies was the small number of patients.

This study has several limitations. First, the number of patients who underwent LLND after preoperative PET was limited. Second, in this study, PET/CT evaluation was performed before the preoperative treatment in patients who underwent chemoradiation therapy or chemotherapy preoperatively. Therefore, the pathological findings were influenced by preoperative treatments. However, the complete response rate to preoperative treatment in this cohort was 7.3%. Moreover, LLN recurrence continues to be a significant problem after chemoradiotherapy plus TME in LLNs with a short axis of at least 7 mm on an MRI scan [12]. Therefore, it is unlikely that LLN metastases had completely disappeared post chemoradiotherapy.

An important point to note is that magnetic resonance imaging (MRI) remains the more useful for local diagnoses than PET-CT. However, PET-CT imaging may be effective for improving the accuracy in preoperative diagnoses, such as diagnoses of distant metastases as well as regional and lateral lymph node metastases. PET-CT, however, is not an alternative to MRI. Third, in this study, there were no cases where the SUVmax was difficult to measure due to bulky primary tumors in cases where lymph nodes were identified by MDCT. However, it is predicted that the diagnosis will be difficult in such cases.

## Conclusions

In present, the appropriate cutoff value of SUVmax in lateral lymph nodes metastasis is not clear.

The diagnosis of the LLN metastases using PET/CT was predicted with the highest sensitivity when the cutoff value of the LLN SUV_max_ was set at 1.5. Therefore, this criterion may be useful in determining indications for LLN metastasis, although a prospective study with a large sample size is warranted for a definitive conclusion.

We will conduct validation study as a prospective study in the future.

## Data Availability

The datasets generated and/or analyzed during the current study are not publicly available, due to the privacy of the enrolled subjects, but these may be requested from the corresponding author, upon reasonable request.

## Abbreviations

AUC: Area under the curve
CT: Computed tomography
^18^F-FDG: ^18^F-fluorodeoxyglucose
FN: False negative
FP: False positive
LLN: Lateral pelvic lymph node
LLND: Lateral lymph node dissection
MDCT: Multi-detector row computed tomography
MRI: Magnetic resonance imaging
NPV: Negative predictive value
PET: Positron emission tomography
PPV: Positive predictive value
ROC: Receiver operating characteristic
ROI: Region of interest
SUV_max_: Maximum standardized uptake value
TME: Total mesorectal excision
